# Comparison of the adhesion and endocytosis of calcium oxalate dihydrate to HK-2 cells before and after repair by *Astragalus* polysaccharide

**DOI:** 10.1080/14686996.2019.1697857

**Published:** 2019-11-26

**Authors:** Jin Han, Da Guo, Xin-Yuan Sun, Jian-Min Wang, Jian-Ming Ouyang, Bao-Song Gui

**Affiliations:** a Department of Nephrology, the Second Hospital of Xi’an Jiaotong University, Xi’an, China; b Institute of Biomineralization and Lithiasis Research, Jinan University, Guangzhou, China

**Keywords:** *Astragalus* polysaccharide, calcium oxalate, cell repair, endocytosis, cell adhesion, molecular weight, 212 Surface and interfaces, 500 Characterization

## Abstract

This work investigated the effects of repairing injured renal proximal tubular epithelial (HK-2) cells by using three *Astragalus* polysaccharides (APS) with different molecular weights and the adhesion and endocytosis of HK-2 cells to the calcium oxalate dihydrate (COD) nanocrystals before and after repair to develop new products that can protect against kidney stones. HK-2 cells cultured *in vitro* were injured with 2.6 mmol/L oxalic acid to establish a damaged cell model. Three kinds of APS (APS0, APS1, and APS2 with molecular weights of 11.03, 4.72, and 2.60 kDa, respectively) were used to repair the damaged cells. The changes in the adhesion and endocytosis of 100 nm COD crystals to cells before and after the repair were detected. After the repair of HK-2 cells by the APS, the speed of wound healing of the damaged HK-2 cells increased, and the amount of phosphatidylserine (PS) ectropion decreased. In addition, the proportion of cells with adhered COD crystals decreased, whereas the proportion of cells with internalized crystals increased. As a result of the repair activity, APS can inhibit the adhesion and promote the endocytosis of COD nanocrystals to damaged cells. APS1, which had a moderate molecular weight, displayed the strongest abilities to repair the cells, inhibit adhesion, and promote endocytosis. Thus, APS, particularly APS1, may serve as potential green drugs for preventing kidney stones.

## GRAPHICAL ABSTRACT

**Figure:**
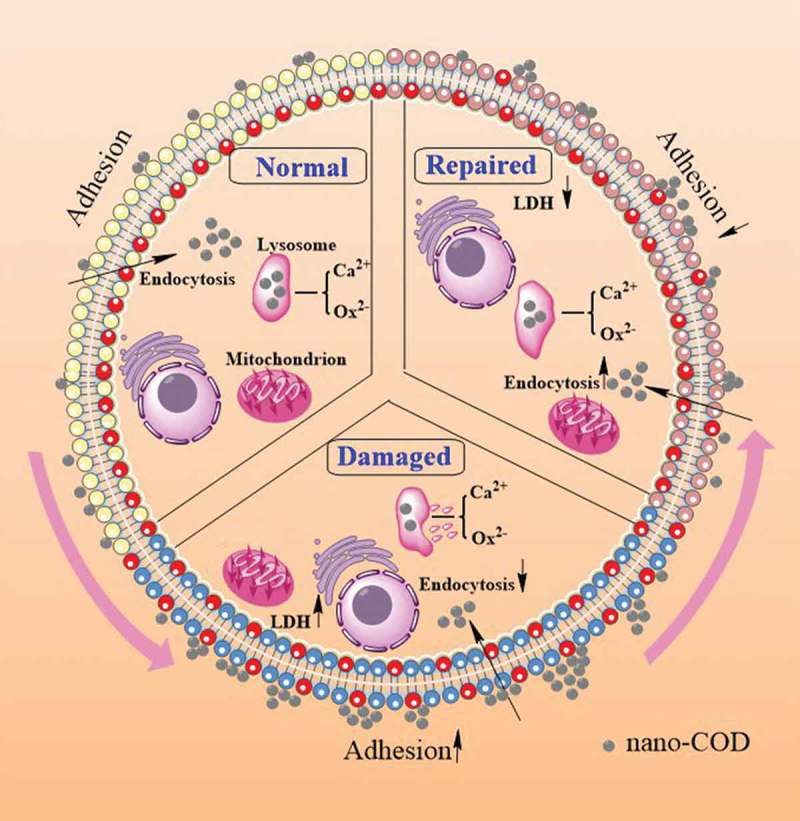

## 1. Introduction

Kidney stone is a global, frequently occurring disease with a high recurrence rate. Approximately 70% of the kidney stones are calcium oxalate (CaOx) stones, including calcium oxalate monohydrate (COM) and calcium oxalate dihydrate (COD) stones [1,2]. Renal epithelial cell damage is a key factor in the formation of stones [3]. Once renal epithelial cells are damaged, the cell surface expresses hyaluronic acid (HA), phosphatidylserine (PS), and other negatively charged molecules [4–6]. These molecules promote the nucleation, aggregation, and adhesion of CaOx crystals, which further damage the cells and aggravate the risk of kidney stone formation [7]. Verkoelen et al. [8] found that the adhesion of COM on the surface of normal Madin Darby canine kidney (MDCK) cells was 0.20 ± 0.03 μg/cm^2^, which increased to 2.3 ± 0.3 μg/cm^2^ after the injury. When the damaged cells self-repaired, the amount of adhered crystals was reduced to 0.16 ± 0.02 μg/cm^2^, which was close to that of normal cells.

The repair of damaged cells inhibits the adhesion of CaOx crystals and therefore reduces the risk of kidney stone formation. De Cógáin et al. [9] found that a polysaccharide extract from *Arabidopsis* (ACE) inhibits not only the COM crystallization but also the adhesion of COM crystals to MDCK cells. The addition of ACE and COM crystals to MDCK-1 cells significantly decreased the crystal adhesion. By contrast, when the MDCK cells were pretreated with ACE for 0.25 or 24 h before COM crystals were added, the crystal adhesion was unaffected by time, indicating that the crystal adhesion was inhibited because the polysaccharide covered the crystal surface and changed the interaction between the crystal and the cell receptor.

CaOx crystals attached to the cell surface can be endocytosed into cells within 30 min under the influence of microvilli [10]. Subsequent endocytic crystals are transferred to lysosomes and dissolved under the action of numerous hydrolytic enzymes to release Ca^2+^ and Ox^2-^ ions. This rapid uptake of crystals adhering onto the cell surface is considered a protective mechanism of cells that eliminates crystals on the cell surface and reduces the risk of kidney stone formation [11,12]. Schepers et al. [11] incubated radiolabeled [^14^C]COM (1.46 mg/mL) with MDCK-II cells. The amount of endocytic crystals in the cells increased from 0.15 ± 0.03 g/10^6^ cells to 3.85 ± 0.04 g/10^6^ cells as the incubation time was prolonged from 30 min to 300 min. The amount of crystals that were swallowed within this period increased linearly with time. However, when the endocytic crystal exceeded the cell’s ability to remove itself, the number of endocytic crystals became positively correlated with the cell injury [13]. The excessive endocytosis of CaOx crystals can cause lysosomal disruption, leading to cell apoptosis or necrosis, thereby increasing the risk of stone formation.

In the literature on CaOx crystals and renal epithelial cells, more studies are available on COM than on COD, even though COD is only the second most common, with a frequency of up to 43% [14]. Previous studies showed that COD can nucleate and adhere to renal tubular epithelial cells [15].

Our previous research [16,17] found that degraded soybean and algal polysaccharides exert a repair effect on damaged renal epithelial cells and can regulate the formation of CaOx crystals. However, limited reports are available on the difference in adhesion and endocytosis of COD crystals to renal epithelial cells before and after repair.

Radix *Astragalus*, the root of *Astragalus* membranaceus, is commonly used in traditional Chinese medicine. *Astragalus* polysaccharide (APS) is considered an important bioactive component of radix *Astragalus* and has negligible side effects. APS displays antioxidant, antitumor, and antiaging properties and protects the cardiovascular system, liver, and kidney [18,19]. The main components of APS are rhamnose, arabinose, xylose, mannose, galactose, and glucose. Given that APS is rich with –COOH negative-charge groups [20], APS maintains the cell surface negative charge and repairs the charge barrier; thus, it may be used to repair damaged renal epithelial cells. However, natural APS has a large molecular weight and a large molecular volume, which hinder its entry into the body across multiple cell membranes to exert its biological properties. Therefore, APS must be degraded.

In our previous study [21], we obtained three degraded APSs, namely, APS0, APS1, and APS2, which had molecular weights of 8.38, 4.72, and 2.60 kDa, respectively. The structures of these polysaccharides were characterized by ^1^H NMR, ^13^C NMR, FT-IR, and GC/MS. Results revealed that the degradation process did not change the main chain structure of APS. The monosaccharides of all APSs consist of rhamnose, arabinose, fucose, sugar, mannose, glucose, and galactose. The main chain is composed of the (1→4) connected Glcp, and the branch point is located at the C-6 position of the (1→6) connected Glcp, both containing 1,4-linked glucuronic acid fragments. The abilities of the three polysaccharides to remove hydroxyl and ABTS radicals and their reducing abilities were closely related to their molecular weight. APS1, which has a moderate molecular weight of 4.72 kDa, exhibited the greatest antioxidant activity.

This study compared the differences in the adhesion and endocytosis of COD nanocrystals between HK-2 cells before and after APS repair to provide an experimental basis for further exploration of the formation mechanism of kidney stones and the development of new anti-stone drugs.

## 2. Experimental methods

### 2.1 Reagents and instruments

The original *Astragalus* polysaccharide (APS0) was provided by Beijing Puboxin Biotechnology Co., Ltd. The polysaccharide content was ≥95%. The degradation of polysaccharides was performed as previously described [16]. The molecular weights of APS0, APS1 and APS2 were 11.03, 4.72 and 2.60 KDa, respectively. Oxalate and other chemical reagents were all analytically pure and purchased from Guangzhou Chemical Reagent Factory of China (Guangzhou, China).

**Figure 1.**
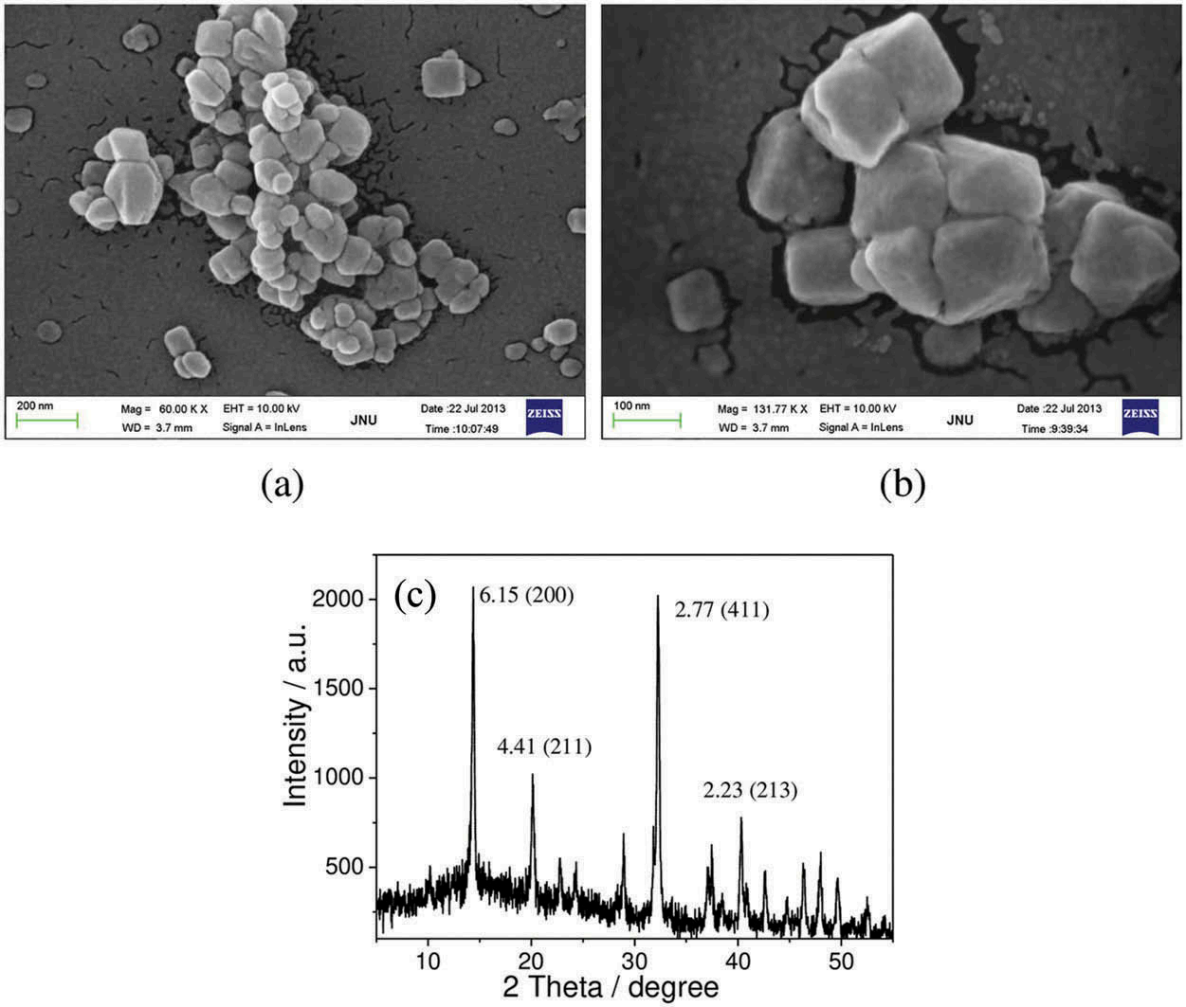
SEM images (a, b) and XRD pattern (c) of nano-COD.

Calcium oxalate dihydrate (COD) was synthesized according to the previous reference [22]. Scanning electron microscopy (SEM) and X-ray diffraction (XRD) indicate that it is a target crystal with a size of 100 ± 15 nm (Figure 1).

Human kidney proximal tubular epithelial (HK-2) cells were purchased from Shanghai Cell Bank, Chinese Academy of Sciences (Shanghai, China). Fetal bovine serum and cell culture medium (DMEM) were purchased from HyClone Biochemical Products Co. Ltd. (Beijing, China). Cell proliferation assay kit (Cell Counting Kit-8，CCK-8) was purchased from Dojindo Laboratory (Kumamoto, Japan). Rabbit anti-mouse IgG conjugated with fluorescein isothiocyanate (FITC-IgG), annexin V-FITC, carbonyl cyanide 3-chlorophenylhydrazone (CCCP) and lactate dehydrogenase (LDH) kit were all purchased from Shanghai Beyotime Bio-Tech Co., Ltd. (Shanghai, China).

The characterization setups included UV-visible spectrophotometer (Cary 500, Varian, USA); Fourier transform infrared absorption spectrometer (EQUINOX 55, Bruker, Germany); microplate reader (SafireZ, Tecan, Switzerland); flow cytometry (FACS Aria, BD, USA); field emission scanning electron microscope (ULTRA 55, ZEISS, Germany); and optical microscope (OLYMPUS, CKX41, Japan).

### 2.2 Cell culture

HK-2 cells were cultured in a DMEM-F12 culture medium containing 10% fetal bovine serum, 100 U/ml penicillin-100 μg/ml streptomycin antibiotics with pH 7.4 at 37°C in a 5% CO_2_ humidified environment. Upon reaching 80–90% confluent monolayer, cells were blown gently after trypsin digestion to form cell suspension for the following cell experiment.

### 2.3 Wound-healing assay

The suspension cells were inoculated in a six-well culture plate at a concentration of 1.5 × 10^5^ cells/mL and 2 mL/well. The samples were added with 10% fetal bovine serum in DMEM and incubated at 37°C for 24 h. The experimental models were divided into three groups: 1) normal group: in which only serum-free culture medium was added; 2) damaged group: in which serum-free culture medium with 2.6 mmol/L oxalate was added and incubated for 3.5 h; 3) repair group: in which serum-free medium containing 60 μg/mL APS0, APS1, and APS2 was added to repair the damaged cells and incubated for 10 h. Once the incubation time was completed, the culture medium was aspirated, and a linear trace with a width of 400 μm was drawn in a certain direction with a sterile 10 μL pipette tip. After washing with PBS twice, fresh culture medium was added to continue the culture. Changes in the scratches were observed under an optical microscope at regular intervals. Photos were captured, and cell healing rate was calculated.

### 2.4 Cytotoxicity measurement of APS on HK-2 cells

Cell suspension with a cell concentration of 1 × 10^5^cells/mL was inoculated into each well in a 96-well plate and incubated for 24 h. Afterward, the culture medium was removed and 100 μL of 60 μg/mL APS with different molecular weights was added. Each group was repeated in five parallel wells. After incubation for 24 h, 10 μL CCK-8 was added to each well and incubated for 1.5 h. The absorbance was measured at 450 nm with an enzyme reader.

### 2.5 Repair effect of APS on damaged HK-2 cells by CCK-8

Cell suspension with a cell concentration of 1 × 10^5^cells/mL was inoculated per well in 96-well plates and incubated in DMEM culture medium for 24 h. The cells were divided into four groups: (1) Control group of background: cell-free culture medium group; (2) Normal control group: in which only serum-free culture medium was added; (3) Damaged group: in which serum-free culture medium with 2.6 mmol/L oxalate was added and incubated for 3.5 h; (4) Repair group: in which the serum-free medium containing 60 μg/mL APS0, APS1 and APS2 was added to repair the damaged cells and incubated for 10 h. After the repair was completed, the OD values were measured using the enzyme mark instrument at 450 nm to detect the repair capacity of polysaccharide.

### 2.6 LDH release before/after polysaccharide repair

Cell suspension with a cell concentration of 1 × 10^5^cells/mL was inoculated per well in 96-well plates and incubated in DMEM culture medium for 24 h. The cells were divided into five groups: (1) cell-free culture medium wells (control wells of background); (2) control wells without drug treatment (sample control wells); (3) cells without drug treatment for the subsequent cleavage of the wells (sample maximum enzyme activity control wells); (4) injury group: the serum-free medium containing 2.6 mmol/L oxalate was added and incubated for 3.5 h; (5) repair group: the serum-free medium containing 60 μg/mL APS0, APS1 and APS2 was added to repair the damaged cells and incubated for 10 h. After the repair was completed, an enzyme mark instrument was used to detect the OD value of each group according to LDH kit method. The results were calculated as:

LDH release amount (%) = (the absorbance of samples treated group–the absorbance of sample control group)/(the absorbance of sample maximum enzyme activity–the absorbance of sample control group) ×100.

### 2.7 Phosphatidylserine (PS) content on the cell surface before/after polysaccharide repair

The suspension cells were inoculated in a 6-well culture plate at a concentration of 1 × 10^5^ cells/mL and 2 mL/well, and 10% fetal bovine serum in DMEM medium was added and incubated at 37°C for 24 h. The experiment model was divided into three groups: 1) normal group, 2) injury group, 3) positive control group, 50 μmol/L CCCP was used to induce PS eversion, and 4) repair group. After the incubation time was reached, the culture medium was aspirated, washed twice with PBS, 100 uL of Binding Buffer and 10 uL FITC-labeled Annexin-V were added, and the cells were light-shielded for 30 min at room temperature. After treatment, the cells were detected by flow cytometry.

### 2.8 Fluorescent labeling of COD crystals

Preparation of FITC-IgG fluorescent-labeled crystals [23]: Pellet the well-weighed crystals, sterilize them by UV for 40 min, prepare them to 400 μg/mL solution with serum-free medium, sonicate for 5 min. A 10 μL FITC-labeled anti-rat IgG (1 mg/mL) was added to 4 mL of DMEM solution with 400 μg/mL COD and incubated overnight at room temperature in the dark. Unreacted free FITC-IgG was dialyzed by dialysis bag (Mw 8000–14000). After removal, the DMEM solution was removed by centrifugation, washed twice with PBS, dried by centrifugation, and stored. The FITC-IgG-labeled crystals were resuspended in anhydrous ethanol, sonicated for 10 min and placed on glass slides, and air-dried and observed under a fluorescence microscope.

### 2.9 Quantitative analysis of the percentage of cells adhered by crystals by flow cytometry

The suspension cells were inoculated in a 6-well culture plate at a concentration of 1 × 10^5^cells/mL and 2 mL/well, and 10% fetal bovine serum in DMEM medium was added and incubated at 37°C for 24 h. Then, the cells were transferred to a 4°C environment for cultivation 30 min to inhibit the endocytic activity of cells, and meanwhile, crystals only can adhere to cells. The medium was then aspirated and washed twice with cold PBS. The experimental models were divided into four groups: 1) blank group: serum-free medium was added; 2) control group: 150 μg/mL freshly labeled COD by green fluorescence was added to normal HK-2 cells; 3) injury group: HK-2 cells after oxalic acid injury were added with freshly prepared 150 μg/mL green fluorescence-labeled COD; 4) repair group: 150 μg/mL freshly prepared green fluorescence-labeled COD was added the cells after the repair of APS, and four groups of cells were transferred to 4°C for 1 h. After the culture time is reached, the culture medium is aspirated and washed twice with cold PBS to remove the unattached and non-tightly adherent crystals. After the trypsin digestion, the cells are resuspended in PBS, and the average fluorescence intensity and adherence are measured by flow cytometer. Cells with FITC signal can be considered as cells with adherent crystals.

### 2.10 Examination of a percentage of the cells that have endocytosed crystals by flow cytometer

The suspension cells were inoculated in a 6-well culture plate at a concentration of 1 × 10^5^cells/mL and 2 mL/well, and 10% fetal bovine serum in DMEM medium was added and incubated at 37°C for 24 h. The culture solution was aspirated and washed twice with PBS. The experimental model was divided into three groups: 1) normal group: serum-free medium was added for 6 h without any reagents; 2) injury group: HK-2 cells after oxalic acid injury were added with freshly prepared 150 μg/mL FITC-IgG labeled COD (100 nm) and cultured at 37°C for 6 h; 3) repair group: freshly prepared 150 μg/mL fluorescently labeled COD was added after repair of APS and cultured at 37°C for 6 h. After reaching the incubation time, the liquid was aspirated and the adherent crystals were removed by complexation with 5 mM EDTA for 5 min, washed with PBS and resuspended. The proportion of fluorescent cells was measured by flow cytometer, and cells at the presence of FITC signal were considered as that cells have endocytosed crystals.

### 2.11 Observation of crystal adhesion on the cell surface by SEM

The suspension cells were inoculated into a 12-well plate covered with coverslips at a cell concentration of 1 × 10^5^cells/mL, with 1 mL per well. After incubation for 24 h, the cells were almost completely covered with coverslips. The cells were then divided into three groups: control group, injury group, and repair group. After injury and repair were completed, all culture fluids on the cells were aspirated and the cells were washed twice with D-Hanks solution. The three groups of cells were added serum-free medium containing a final concentration of 150 μg/mL COD. After 6 h of incubation, the supernatant was aspirated. After washing the cells twice with PBS, 2.5% glutaraldehyde was added for 24 h and then washed with PBS three times. Dehydrated by graded ethanol (30%, 50%, 70%, 90%, 100%), dried at the critical point of CO_2_, and sprayed with gold. After the sample was processed, SEM was used to observe crystal adhesion on the cell surface.

### 2.12 Statistical analysis

Experimental data were expressed as mean ± SD from at least three independent experiments. The experimental results were statistically analyzed using SPSS 13.0 software and a Tukey test was used to analyze the differences between the mean of each experimental group and control group. P < 0.05 was considered statistically significant, indicating a significant difference, P < 0.01 indicating a very significant difference, P > 0.05 indicates no significant difference.

## 3. Results

### 3.1 Polysaccharides promote wound healing

**Figure 2.**
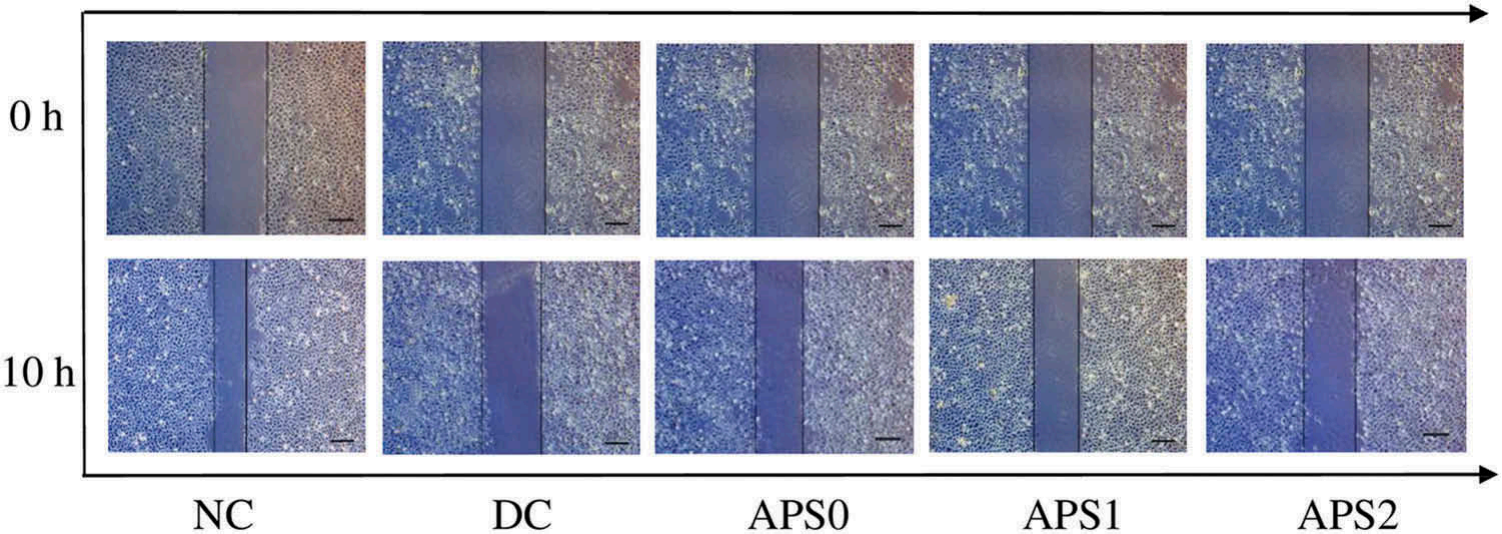
Effects of APS with different molecular weight on the wound healing of HK-2 cells. NC: normal control, DC: damaged control; polysaccharide concentration: 60 μg/mL; oxalate damage concentration: 2.6 mmol/L; damage time: 3.5 h; repair time: 10 h, scale bar: 150 μm.

To study the effect of APS of different molecular weights on the wound healing of HK-2 cells, we performed a wound-healing assay, as shown in Figure 2. In the control group, the scratch distance was reduced after 10 h of cell culture, and the cells in the scratch center were connected, indicating that the control group had the fastest wound-healing rate (18.7 ± 1.1 μm·h^−1^, Table 1). By contrast, the injury group healed very slowly, and its healing rate was only 1.5 ± 1.1 μm·h^−1^. After the damaged cells were repaired with APS0, APS1, and APS2, the wound-healing rates became higher than that in the injury group but lower than that in the control group. Among the three repair groups, the APS1 repair group demonstrated the fastest healing rate (10.3 ± 1.1 μm·h^−1^). The speed of scratch healing followed the order of control group > APS1 group > APS0 group > APS2 group > injury group.

**Table 1.** Wound-healing rates within 10 h of HK-2 cells after polysaccharides repair.

Group	NC	DC	APS0	APS1	APS2
Wound-healing distance (L)/μm	197.4 ± 0.8	24.5 ± 0.5	104.7 ± 1.3	113.4 ± 1.1	56 ± 2
Wound-healing rate (*v*)/μm·h^−1^	18.7 ± 1.1	1.5 ± 1.1	9.5 ± 1.1	10.3 ± 1.1	4.6 ± 1.2

NC represents normal control group; DC is 2.6 mmol/L oxalate damage control group (damaged control), polysaccharide concentration is 60 μg/mL, damage time: 3.5 h, repair time: 10 h.

The repair and proliferation of cells by polysaccharides are important in wound healing. Many studies have confirmed that polysaccharides can promote cell proliferation [24,25]. Polysaccharides can stimulate surviving cells in the wounded nephron to migrate along the basement membrane, thereby rapidly restoring tubular structure and function [26]. Given that normal renal epithelial cells can effectively prevent crystal adhesion, a repair group with a fast healing rate can reduce the risk of stone formation.

### 3.2 Polysaccharides improve cell viability

**Figure 3.**
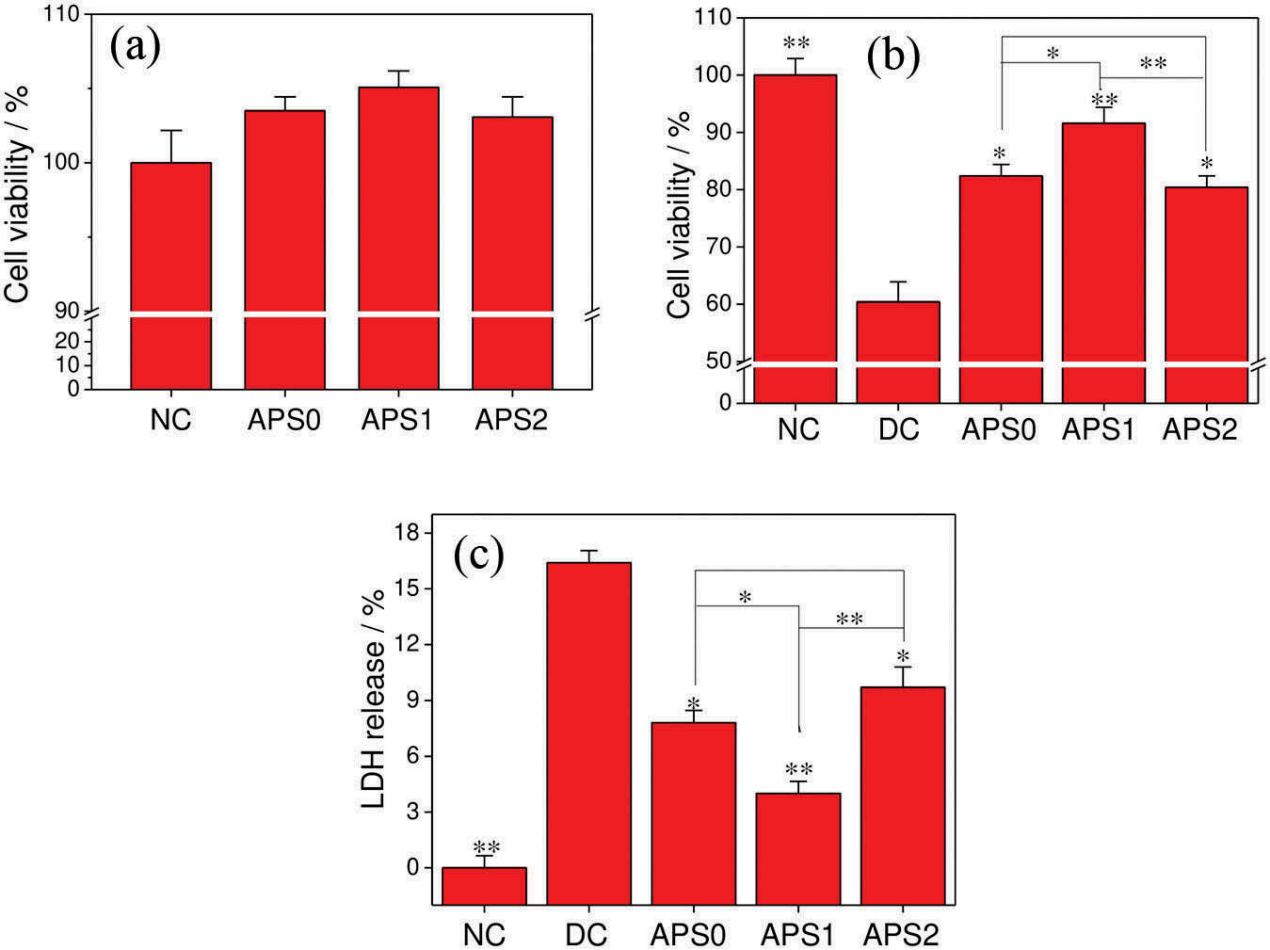
Cytotoxicity of APSs with different molecular weights on HK-2 cells (a). Changes in cell viability (b) and LDH release (c) of injured HK-2 cells after APS repair. NC: normal control, DC: damaged control; polysaccharide concentration: 60 μg/mL; oxalate damage concentration: 2.6 mmol/L; damage time: 3.5 h; repair time: 10 h. Compared with DC group, *P < 0.05; **P < 0.01.

The cytotoxicity of APS0, APS1, and APS2 with molecular weights of 11.03, 4.72, and 2.61 kDa, respectively, on HK-2 cells was evaluated by CCK-8 method (Figure 3(a)). When HK-2 cells were treated with 60 μg/mL APSs for 24 h, the cell viabilities were all above 100%. This result indicated that at this concentration, the three APSs were nontoxic and had promoting effects on HK-2 cells.

The repair effects of APS0, APS1, and APS2 on damaged HK-2 cells were compared at a concentration of 60 µg/mL (Figure 3(b)). After repair, the viability of the damaged HK-2 cells increased from 60% ± 4% (damaged group) to 82% ± 2% (APS0), 92% ± 3% (APS1), and 80% ± 2% (APS2). The cell viabilities of all repair groups were greater than that of the damaged group and lower than that of the control group (100% ± 3%). The molecular weight of the polysaccharide affected its repair effect. APS1 showed the best repair effect.

### 3.3 Polysaccharides inhibit lactate dehydrogenase (LDH) release

Under normal circumstances, LDH is located in the cytoplasm. The cells were attacked by foreign substances, which destroy the cell membrane structure, resulting in the release of LDH in the cytoplasm to the culture fluid. When the LDH content in the culture fluid increased, the damage degree of the cell membrane increased, and the cell membrane integrity deteriorated [27].

Figure 3(c) shows the changes in the LDH release of damaged HK-2 cells after repair by the three APSs. Compared with that in the injury group, the release of LDH in the repair group cells (4–9.7%) decreased to varying degrees, indicating that the three APSs exerted a repairing effect on the damaged HK-2 cells. APS1 with moderate molecular weight displayed the best repair effect.

### 3.4 Polysaccharides decrease PS eversion

In general, PS is located inside the cell membrane. At the early stage of apoptosis, PS can be turned from the inner side to the surface of the cell membrane. Phospholipid-binding protein V (annexin V) is a calcium-dependent phospholipid-binding protein that has a high degree of binding with PS. Therefore, annexin V can be used as a probe to detect the content of PS exposed on the cell membrane [5].

**Figure 4.**
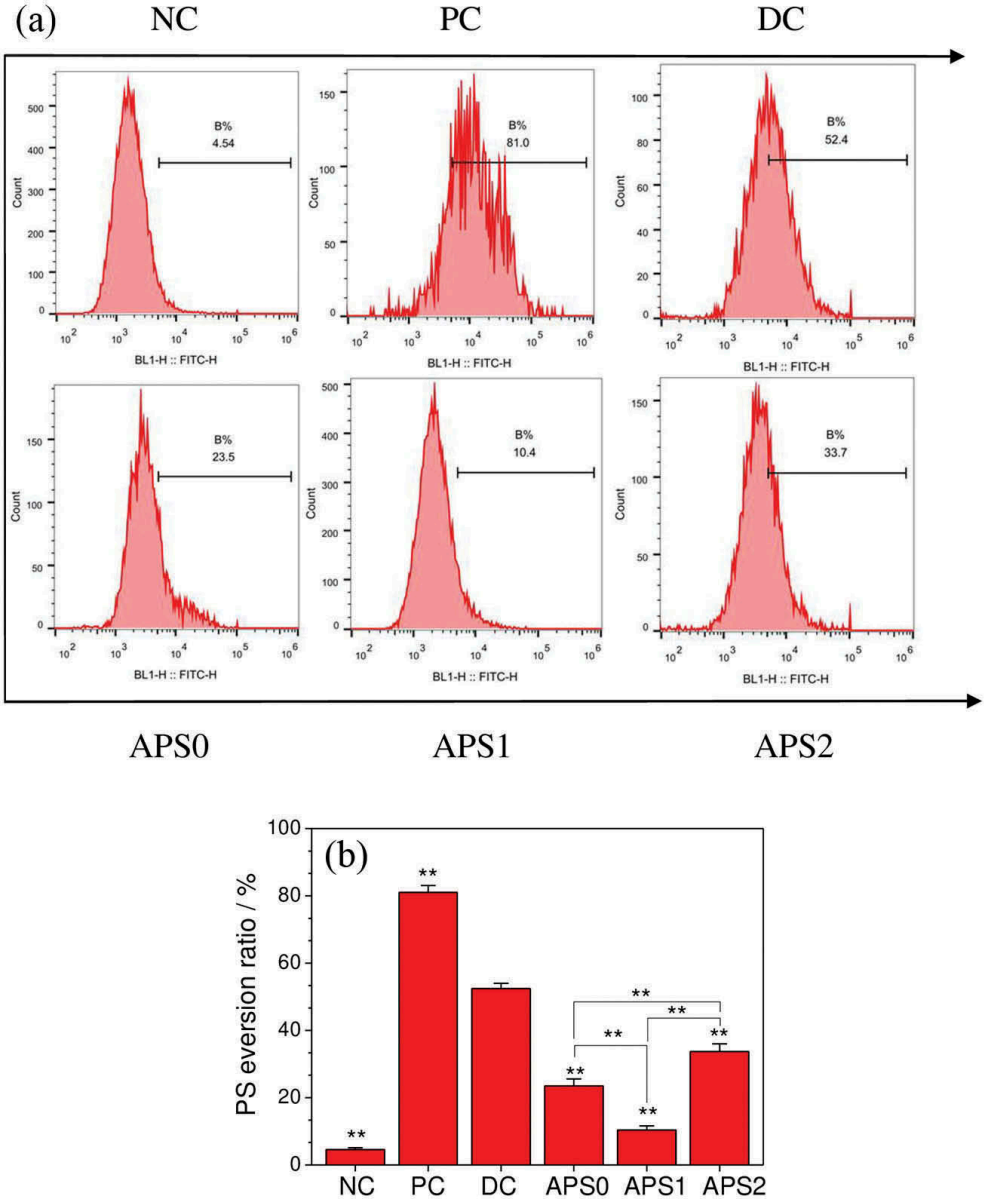
Quantitative detection of PS eversion of damaged HK-2 after APS repair by flow cytometry. (a) Histogram of PS eversion ratio; (b) statistical histogram of the percentage of PS eversion. NC: normal control, PC: positive control (CCCP); DC: damaged control; polysaccharide concentration: 60 μg/mL; oxalate damage concentration: 2.6 mmol/L; damage time: 3.5 h; repair time: 10 h. Compared with DC group, **P < 0.01.

Figure 4 shows the changes in PS eversion in HK-2 cells before and after repair. The PS eversion in the control group (4.54%) was significantly lower than those in the injury (52.4%) and positive control (81.0%) groups. After being repaired with APS0, APS1, and APS2, HK-2 cells had PS eversion of 23.5%, 10.4%, and 33.7%, respectively. APS inhibited the occurrence of PS eversion in HK-2 cells. After the reduction of PS eversion, the ability of PS to adhere to CaOx crystals was weakened, thereby inhibiting the formation of kidney stones.

### 3.5 Polysaccharides inhibit the adhesion of COD nanocrystals to cells

**Figure 5.**
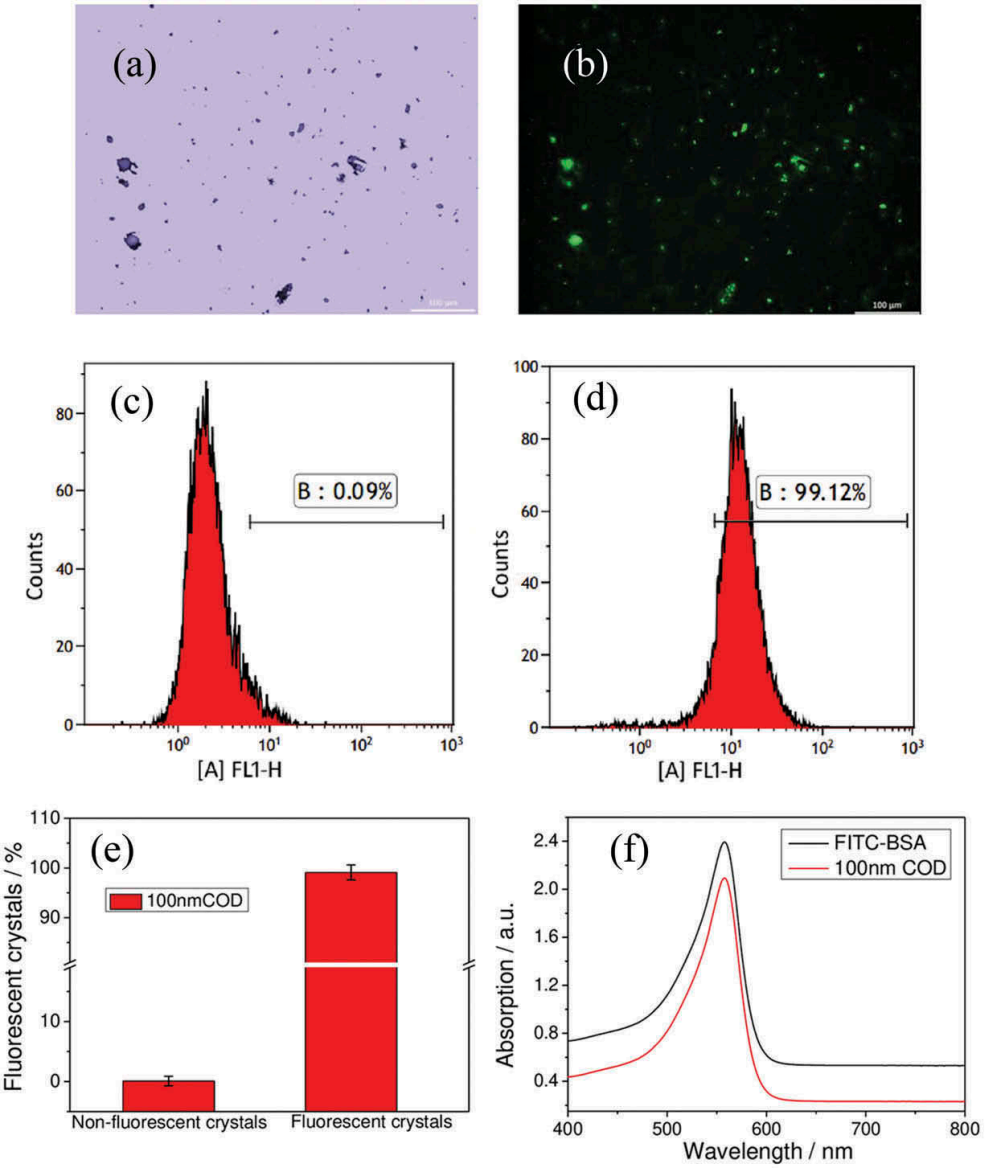
FITC-IgG fluorescent labeling of nano-COD crystals. (a, b) COD crystal images before and after labeling; (c, d) flow cytometric analysis histogram of fluorescent ratio of plain and FITC-IgG fluorescent-labeled nano-COD crystal; (e) fluorescent COD crystal percentage statistics histogram; (f) UV-visible absorption spectra of FITC-IgG and its labeled COD solution.

COD crystals can be fluorescently labeled with FITC-IgG to show green fluorescence under a fluorescence microscope. As shown in Figure 5(a,b), COD crystals had a uniform morphology before and after the fluorescent label, indicating that the label did not affect the COD crystals. In addition, more than 99% of the crystals can be labeled with the fluorescent molecule (Figure 5(d)). The UV-visible spectra revealed that the fluorescent-labeled COD crystals had a characteristic FITC absorption peak at 488–500 nm (Figure 5(f)), indicating that the labeled COD crystal signal can be detected by flow cytometry and microplate readers. In view of the aggregation phenomenon of nano-COD in serum-free DMEM, the apparent size of the particles in Figure 5(a,b) was larger than 100 nm, similar to that in the literature [23].

**Figure 6.**
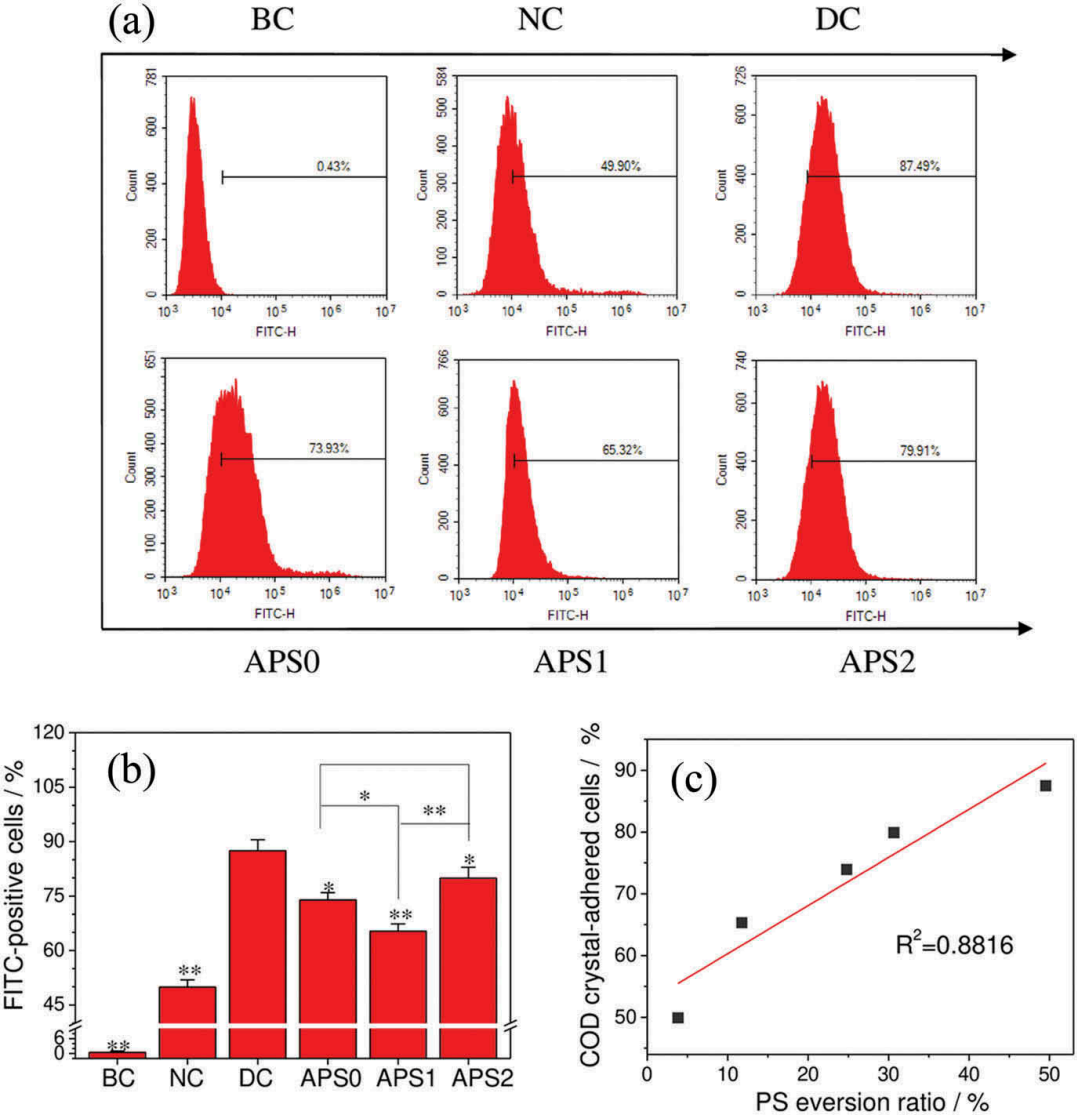
Quantitative detection of the proportion of adherent nano-COD crystals of HK-2 cells before and after APS with different molecular weights repair by flow cytometry. (a) Histogram of the proportion of cells with adhered COM crystals; (b) statistical histogram of the proportion of cells with adhered COM crystals; (c) the linear relationship of a percentage of cells adhering to nano-COD crystals and PS expression, *R*^2^ is a linear correlation coefficient. BC: blank control, NC: normal control, DC: damaged control; Polysaccharide concentration: 60 μg/mL; oxalate damage concentration: 2.6 mmol/L; damage time: 3.5 h; repair time: 10 h. Compared with DC group, *P < 0.05; **P < 0.01.

The endocytosis of COD crystals to cells was inhibited at 4°C, and crystal adhesion was unaffected [28]. Therefore, the cells that were detected with a FITC signal by flow cytometry can be considered the cells which had COD crystals adhered. As shown in Figure 6(a), the non-fluorescent-labeled COD crystals incubated with the cells for 6 h showed only 0.3% of the cells with fluorescence, indicating that the crystal markers were successful. The proportion of injury group cells that adhered to the COD crystals (87.49%) was significantly greater than that in the control group (49.90%).

Given that the surface microstructure and polarity of the cells changed significantly after damage, several acid-rich membrane components (such as PS) migrated from the inside of the cell membrane to the outside (Figure 4). The apical membrane surface expressed various negative ions that can attract Ca^2+^ ions and calcium oxalate crystals, such as hyaluronic acid (HA), collagen, and osteopontin (OPN) [4,6]. These negatively charged substances were distributed on the surface of the cell membrane and served as sites for the adsorption of Ca^2+^ ions and positively charged COD crystallites that promoted the adhesion of COD crystals.

After APS0, APS1, and APS2 repair, the proportions of cells that adhered crystals were 73.93%, 65.32%, and 79.91%, respectively. The ability of APS1, which had the strongest repairability, to inhibit nano-COD adhesion was also the strongest.

### 3.6 SEM observation of COD nanocrystals adhered to HK-2 cells before and after repair

**Figure 7.**
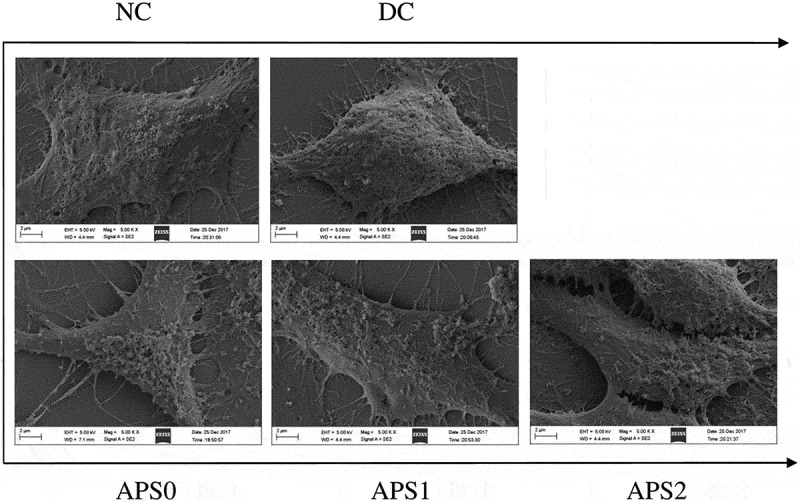
SEM observation of HK-2 cells adherent nano-COD crystals before and after APS repair. NC: normal control, DC: damaged control; polysaccharide concentration: 60 μg/mL; oxalate damage concentration: 2.6 mmol/L; damage time: 3.5 h; repair time: 10 h.

The inhibitory effect of APS on COD adhesion was confirmed by SEM (Figure 7). The amount of adhered crystals on the cell surface of the injury group was greater than that of the control group. The amount of crystal adhesion of each polysaccharide repair group was between that of the injury and control groups. APS1, which had the strongest ability to repair damaged cells, had the strongest ability to inhibit nano-COD adhesion. APSs inhibited the adhesion of calcium oxalate crystals and may hinder the formation of kidney stones.

### 3.7 Polysaccharides promote COD nanocrystal endocytosis in HK-2 cells

**Figure 8.**
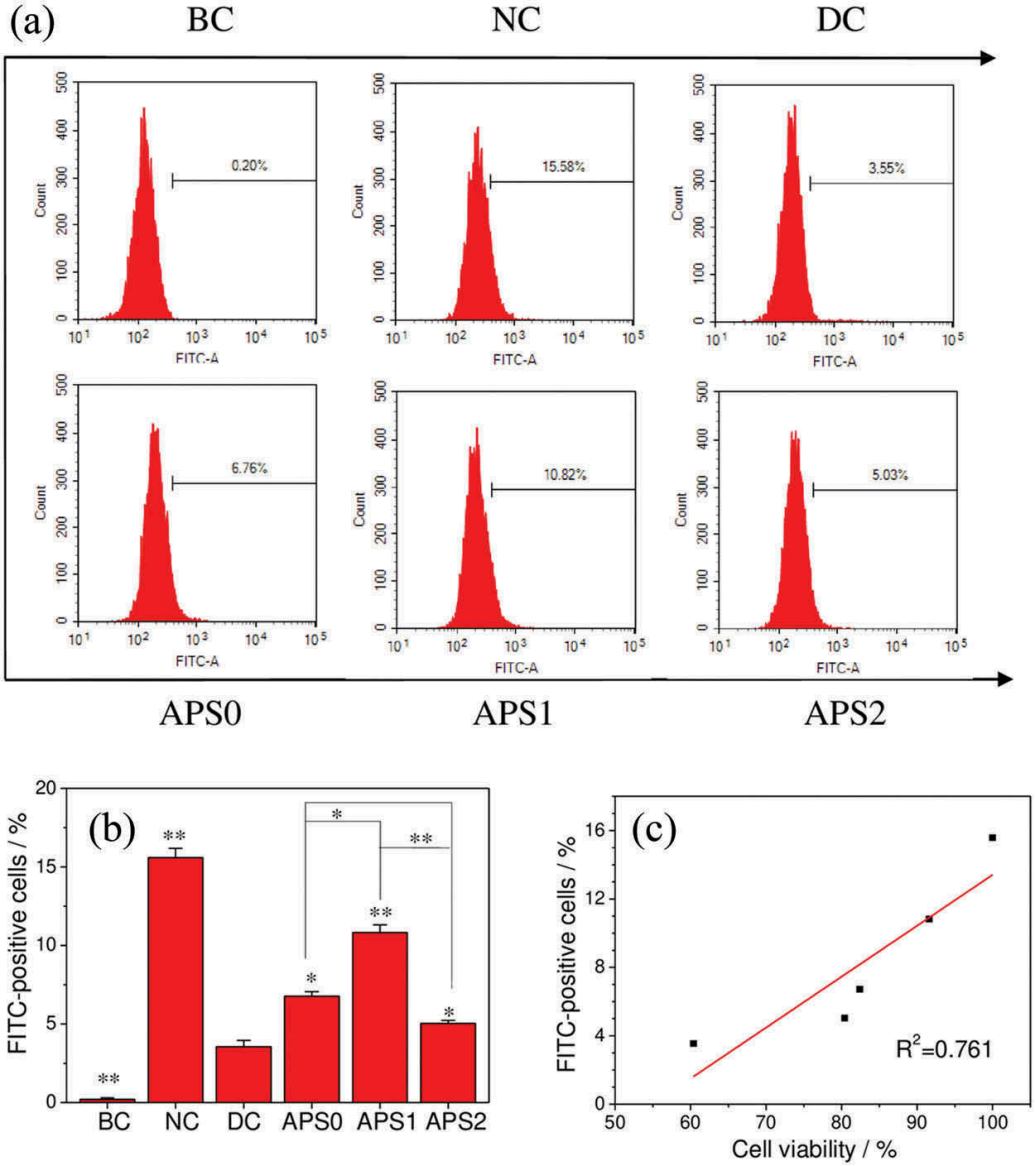
Quantitative detection of endocytotic nano-COD ratio in HK-2 cells before and after APS repair by flow cytometry. (a) Histogram of the proportion of cells with endocytosed COD crystals; (b) statistical histogram of the proportion of cells with fluorescence (i.e. endocytic crystal); (c) the linear relationship between the percentage of endocytosed nano-COD crystals and cell viability. *R*^2^ is a linear correlation coefficient. BC: blank control, NC: normal control, DC: damaged control; polysaccharide concentration: 60 μg/mL; oxalate damage concentration: 2.6 mmol/L; damage time: 3.5 h; repair time: 10 h. Compared with DC group, *P < 0.05; **P < 0.01.

Endocytosis is the process of transporting extracellular substances into cells through the deformation of the plasma membrane [29]. Fluorescent-labeled nano-COD can be used to observe cell endocytosis [30]. As shown in Figure 8, the proportions of cells with endocytosed COD crystals were 15.58% in the control group and 3.55% in the injury group. After being repaired by APS0, APS1, and APS2, the proportion of endocytic cells was between that of the control and injury groups (5.03–10.82%), and the APS1 group had the highest proportion of cells with endocytosed crystals (10.82%). This finding suggested that APS1, which had the highest ability to repair, can also increase the ability of cells to endocytose crystals.

## 4. Discussion

In this study, we investigated the adhesion and endocytosis of COD nanocrystals in HK-2 cells before and after APS repair. We focused on the differences in the adhesion and endocytosis of COD crystals to injured and repaired cells. The results showed that the repair effect of APS can inhibit the adhesion and promote the endocytosis of COD nanocrystals in HK-2 cells.

### 4.1 Relationship between the molecular weight and repairability of APS

The repair effect of APS on HK-2 cells was related to its molecular weight. In terms of repair capacity, the APSs followed the order of APS1 > APS0 > APS2.

1. When the molecular weight was excessively large, the polysaccharide had a compact molecular structure, a large volume, and low water solubility [31]; as such, it cannot easily exert its biological effects through cell membrane disorders. Lei et al. [32] showed that yeast glucans with molecular weights of 12.9, 16.5, and 19.2 kDa have antioxidant activity and immunomodulatory activity *in vitro*. Glucan with larger molecular weight exerts lower activity.
2. When the molecular weight of the polysaccharide was excessively small (such as APS2 with a molecular weight of 2.60 kDa), its biological activity was reduced due to the difficulty in forming a biologically active polymeric structure [33]. Sheng et al. [34] obtained four polysaccharides with molecular weights of 14,528, 12,370, 11,548, and 6403 Da, respectively, by degrading the polysaccharide of *Athyrium multidentatum* (Doll.) Ching; the DPPH radical scavenging rates decreased with decreasing molecular weight.
3. Polysaccharides with a moderate molecular weight (such as APS1 with a molecular weight of 4.72 KDa) not only had higher degrees of freedom and less steric hindrance [35] but were also more easily absorbed by the cell membranes through electrostatic interactions, allowing the cells to be repaired to the maximum. Therefore, degrading polysaccharides from a high molecular weight to an appropriate molecular weight significantly increases their biological activity. Meng et al. [36] showed that the activity of scavenging hydroxyl radicals of hirsute polysaccharides with a molecular weight of 10–20 kDa was better than that with molecular weights of 43.0 and 4.7 kDa.

### 4.2 Relationship between adhesion and kidney stone formation

When the renal tubular epithelial cells were damaged, not only PS eversion to the cell membrane surface (Figure 8) but also PS can be concentrated on the cell surface through a lateral movement in a specific area, thereby providing an effective position for adsorbing Ca^2+^ ions and adhering calcium oxalate crystals. This phenomenon increases the nucleation and adhesion of CaOx crystals and promotes the early formation of kidney stones [26].

The results revealed that the amount of PS eversion increased in injured cells (Figure 4). Therefore, the proportion of cells adhering to the crystals (87.49%) was significantly greater than that in the control group (49.90%). The percentage of cells adhering to crystals in each group was positively correlated to the PS expression (Figure 6(c)).

### 4.3 Relationship between endocytosis and kidney stone formation

Endocytosis is a self-protective mechanism of renal epithelial cells. Endocytosed crystals are transferred to lysosomes and dissolved under the action of numerous hydrolytic enzymes to release Ca^2+^ and Ox^2-^ ions, which are no longer toxic to cells [13]. Therefore, the endocytosis of crystals by renal epithelial cells can effectively reduce the amount of adherent crystals and inhibit the formation of kidney stones.

Chaiyarit et al. [12] confirmed by fluorescence imaging and flow cytometry that after the incubation of FITC-labeled COM (1000 μg/mL) with MDCK for 1 h, the percentage of cells with endocytosed crystals was 14.8% ± 0.9%. As they further tracked the endocytosed crystals for 48 h, they observed that the size of the endocytosed crystals gradually decreased over time, indicating that the endocytosed crystals were slowly dissolved.

These results indicated that the ability of HK-2 cells to endocytose crystals was correlated with the cell viability. The viability of the injured cells decreased, their endocytic capacity was weakened, and the percentage of cells in the endocytic crystals was only 3.55%, which was significantly smaller than that in the control group (15.58%). The percentage of cells with endocytosed crystals after APS repair (5.03–10.82%) was between that of the control and injury groups. Figure 8(c) shows that the percentage of cells with endocytosed crystals of each group was positively correlated with cell viability.

**Figure 9.**
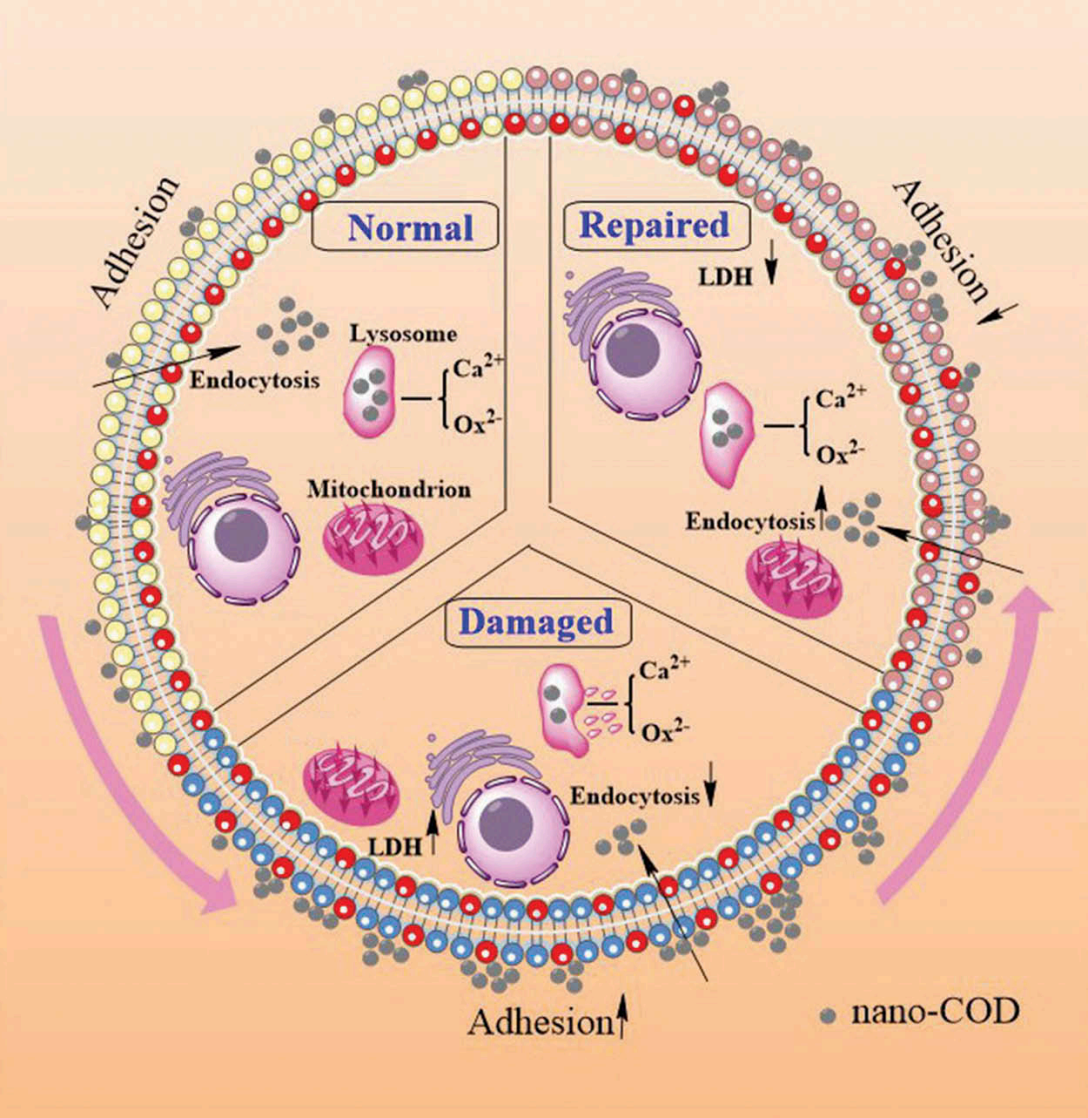
Model of adhesion and endocytosis of nano-COD to damaged HK-2 cells before and after repair by APS with different molecular weights repair.

Figure 9 depicts a model diagram of the adhesion and endocytic nano-COD of damaged HK-2 cells before and after repair by APSs of different molecular weights. Compared with the control group, the PS eversion of the injury group increased, the LDH release increased, the percentage of cells adhering to the crystals increased, the adhered crystals were easily agglomerated, and the percentage of the endocytic crystal cells decreased. Compared with the injury group, the PS eversion of the repair group was reduced, the LDH release decreased, the percentage of cells adhering to the crystals decreased, and the percentage of the endocytic crystal cells increased. The APS repair group cells can reduce the adhesion of calcium oxalate, promote cell endocytosis to the crystal, eliminate adherent crystals, and reduce the risk of kidney stone formation.

### 4.4 Mechanism of action of APS on cell repair, adhesion and endocytosis

In our previous study [21], we verified that the antioxidant ability of APSs *in vitro* (removing hydroxyl and ABTS radicals and reducing ability) and on cells (superoxide dismutase and malondialdehyde generation) is closely related to their molecular weight. APS2, which had moderate molecular weight, showed the highest antioxidant activity and the highest ability to repair HK-2 cells. APSs repaired HK-2 cells by increasing the antioxidant capacity of the cells to block or inhibit cell injury. Cheng et al. [37] reported that *Hericium erinaceus* polysaccharides can scavenge DPPH free radical, improve cell viability, reduce ROS generation, inhibit the reduction of mitochondrial membrane potential, and exhibit protective effects on amyloid beta-induced neurotoxicity. Kim et al. [38] revealed that *Psidium guajava* leaf polysaccharides can relieve H_2_O_2_-induced oxidative stress and DNA injury by scavenging radicals in Vero cells.

The damage of renal epithelial cells promoted the expression of adhesion molecules, such as PS (Figure 4), OPN, and HA [27], aggravating the adhesion of crystals on the cell surface. Xi et al. [39] showed that high calcium-induced cell damage in urine led to OPN overexpression, stimulating the adhesion of CaOx crystals to normal rat renal tubular epithelial cells. APS can repair oxidatively damaged HK-2 cells and can inhibit PS eversion, finally reducing crystal adhesion.

The endocytosis ability of HK-2 cells to nano-COD is positively correlated with cell viability, i.e. greater cell viability results in more crystals that will be endocytosed. Endocytosis is an active transportation process that consumes a large amount of energy [40]. Cells with good cell vitality have a strong metabolism. Therefore, engulfing the crystals attached to the cell surface and reducing the damage of adhered crystal on the cell surface are easier. APSs can improve cell state, promote cell proliferation, thereby inhibiting crystal adhesion and increasing endocytosis.

## 5. Conclusions

Three kinds of APSs (APS0, APS1, and APS2) with molecular weights of 11.03, 4.72, and 2.60 KDa, respectively, repaired HK-2 cells that were damaged by oxalic acid oxidation. Compared with the injury group, the wound-healing rates of the repair group cells increased, the expression of PS on the cell surface decreased, the adhesion onto the COD nanocrystals decreased, and the amount of endocytic crystals increased. The ability of the cells to inhibit crystal adhesion and increase crystal endocytosis after polysaccharide repair was positively correlated with the repairability of the three polysaccharides. The repairability of APS1, which had a moderate molecular weight, was greater than that of APS0 (excessively large molecular weight) and APS2 (low molecular weight). Therefore, APS, particularly APS1, can serve as novel anti-stone polysaccharide drugs.
